# IGF2BP2 promotes pancreatic carcinoma progression by enhancing the stability of B3GNT6 mRNA via m6A methylation

**DOI:** 10.1002/cam4.5096

**Published:** 2022-07-31

**Authors:** Pei Cao, Yufan Wu, Ding Sun, Weigang Zhang, Junyi Qiu, Zuxiong Tang, Xiaofeng Xue, Lei Qin

**Affiliations:** ^1^ Department of General Surgery The First Affiliated Hospital of Soochow University Suzhou Jiangsu China

**Keywords:** B3GNT6, epigenetic modification, IGF2BP2, pancreatic carcinoma, RNA methylation

## Abstract

**Background:**

Pancreatic carcinoma (PC) is a highly lethal cancer with an increasing mortality rate, its five‐year survival rate is only approximately 4%. N6‐methyladenosine (m6A) modification is the most common posttranscriptional modification of RNA, it could affect tumor formation by regulating m6A modifications in the mRNA of key oncogenes or tumor suppressor genes. However, its role in PC remains unclear.

**Methods:**

We combined bioinformatic analysis with in vitro and in vivo experiments to investigate the expression profile of methylation modulators and identify key m6A regulators in the progression of PC. Further study focused on exploring the target genes binding to the regulators through RIP and immunofluorescence staining experiment.

**Results:**

TCGA and Gene Expression Omnibus (GEO) analyses revealed an overall increasing trend in the expression of m6A regulators in PC, and consensus clustering analysis of m6A modification showed that the expression of regulators was negatively correlated with the survival rate. LASSO‐Cox regression analysis revealed that IGF2BP2, METTL3, ALKBH5 and KIAA1429 were associated with hazard ratios (HR), but only IGF2BP2 was sufficiently appropriate for the m6A survival prognosis model. The IHC and WB results verified high protein expression of IGF2BP2 in PC, and IGF2BP2 knockdown inhibited the proliferation and migration of PC cells. We predicted and verified B3GNT6 was observably regulated by IGF2BP2 via RIP assays. In addition, IF staining confirmed the co‐expression of IGF2BP2 and B3GNT6. The tumor‐promoting effect of IGF2BP2 and its co‐expression with B3GNT6 were verified in an animal model.

**Conclusions:**

Elevated m6A levels promote PC progression. IGF2BP2 is a credible marker and modulates B3GNT6 mRNA stability, indicating that IGF2BP2 is a potential prognostic marker and therapeutic target in PC progression.

## Graphical abstract

1. Bioinformatics reveals that m6A regulators are generally elevated in PC. 2. IGF2BP2 enhances the stability of B3GNT6 mRNA via m6A methylation. 3. IGF2BP2 plays a carcinogenic role, promoting tumor formation in vivo.

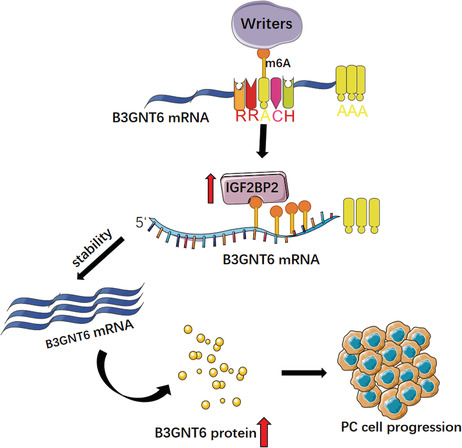

## 1 BACKGROUND

Pancreatic carcinoma has long been one of the most devastating and highly malignant tumors, the five‐year survival rate is only 4%.^1^ In both the United States and China, the morbidity and mortality of PC are less than satisfactory.^2^ Currently, the most common and effective treatment is surgery combined with radiotherapy and chemotherapy, but this regimen exhibits little therapeutic effect.^3^ Considering that PC is characterized by a strong interstitial hyperplastic reaction around cancer cells,^4^ its drug resistance and early invasive metastasis control the high mortality of PC patients.^5^ During the progression of PC, different signaling pathways mediate cancer typing, growth, proliferation and invasion, which makes the tumor microenvironment more complex.^6^

It is well known that RNA is subject to a variety of internal modifications, among which N6‐methyladenosine (m6A) methylation is the most abundant and conserved epigenetic chemical modification.^7^ Scientists have found that m6A methylation is a dynamic reversible process mediated by m6A methylation‐related proteins.^8^ m6A methyltransferase “Writers” (METTL3/14, WTAP) facilitate the installation of methyl groups on RNA, and its demethylation is mediated by “Erasers” (FTO and ALKBH5), then m6A could be recognized by “Readers” (YTHDF1/2/3, IGF2BP1/2/3 and YTHDC1/2) to perform related functions,^9^ including the regulation of mRNA stability,^10^ transcription and translation efficiency,^11^ RNA processing events^12^ and miRNA maturation. It has been found that m6A mRNA methylation plays an essential role in the occurrence and development of tumors,^13^, ^14^ including hepatoblastoma,^15^ bladder cancer^16^ and gastric cancer.^17^ However, research on m6A in PC is still limited. It has been found that WTAP is highly expressed in PC and associated with clinicopathological features, but related mechanisms have not been reported.^18^ IGF2BP2, a member of the IGF2 mRNA‐binding protein family, binds to the 5' UTR of the insulin‐like growth factor 2 (IGF2) mRNA and regulating its translation. It is overexpressed and promotes tumor progression in a variety of cancers. Xu revealed the oncogenic role of IGF2BP2 in PC and could promote cancer proliferation by activating the PI3K/Akt signaling pathway.^19^ Research has found it could regulate DANCR in PC through m6A modification to promote stemness‐like properties and pathogenesis.^20^ Since little is known about IGF2BP2 and m6A methylation in PC. It is urgent to identify the regulation of m6A in PC. Here we classified PC patients according to the overall expression pattern of m6A RNA methylation regulators in the Cancer Genome Atlas (TCGA) database and constructed a risk score based on the least absolute shrinkage and selection operator. We identified a crucial target IGF2BP2, which mediates the malignant progression of PC, promotes B3GNT6 mRNA stability, contributes to further progression of PC.

## 2 METHODS

### 2.1 Downloading of the PC dataset in the cancer genome atlas (TCGA) and gene expression omnibus (GEO)

We used the R package TCGAbiolinks to download the PC transcriptome data from TCGA database and combined them with healthy human tissue transcriptome data in GTEx to explore differential gene expression in PC. Also, RNA sequencing data was downloaded in GEO to measure the expression of regulators in PC. Patients with rational survival data and clinicopathological characteristics were included in the analysis. For group analysis, the median value was used as the cut‐off site.

### 2.2 Clinical bioinformatic analysis

We employed the R software package ConsensusClusterPlus to divide 176 PC patients into two subtypes (PA1 and PA2) according to the expression of the regulators and used principal component analysis (PCA) to evaluate the difference in the gene expression distribution between the two clusters. Additionally, we used the R software package limma to conduct differential gene expression analysis on the subgroups, where fold change (FC) > 2 and *p* < 0.05 were used as the cut‐off values for DEGs. KEGG and GO enrichment analyses were performed with the above DEGs. The risk signature was calculated using the LASSO‐Cox regression algorithm according to the minimum criteria. The risk score was calculated using the formula, where Coefi is the coefficient:

$$\text{Risk score}=\sum_{i=1}^{n}\mathrm{Coe}f_{i}*x_{i}$$

### 2.3 Prediction of IGF2BP2 target genes

To predict and verify the possible downstream targets regulated by IGF2BP2, we analyzed and compared the differential genes between the two subgroups (PA1 and PA2), and the differential genes in PC. In addition, the CLIP dataset in m6A2Target database was used to filter the downstream mRNAs of IGF2BP2, the possible targets of IGF2BP2 were then obtained by intersection, and the follow‐up experiments were carried out to verify it.

### 2.4 Clinical PC specimens

PC together with counterpart adjacent normal tissue samples were obtained from department of General Surgery, the First Affiliated Hospital of Soochow University. The research was ratified by the Ethical Committee of the first affiliated hospital of Soochow University and written informed consent were successively obtained from all participants before the study.

### 2.5 Cell culture

Human CFPAC1 and PANC1 PC cells were purchased from GenePharma Technology Co., Ltd. (Suzhou, China) and used in our research. These cells were cultured in Dulbecco's modified Eagle's medium supplemented with 10% foetal bovine serum (FBS) in an incubator with a 5% CO_2_ atmosphere at 37°C. All cells were free of bacterial and mycoplasma contamination.

### 2.6 Transfection

The target mRNA interference fragments and the NC construct si‐NC were synthesized (Synbio Technologies, China) and co‐transfected with Lipofectamine 2000 reagent (Invitrogen, USA) to knock down gene expression in PC cells; their sequences are shown in Table S1.

### 2.7 Quantification of overall m6A RNA methylation

We used an EpiQuik m6A RNA Methylation Quantification Kit (colorimetric) (Epigentek, USA) to quantify the overall m6A methylation level in PC cells. First, we extracted total RNA and approximately 200 ng RNA was separated as an initial input. Then, the sample RNA, standard positive/negative control and binding solution were bound to the wells respectively for 1 h to assay and capture RNA according to the manufacturer's instruction. After washing, the detection antibody and enhancer solution were added, and color developer solution and stop solution was added prior to measurement of the absorbance. The values were calculated using linear regression equations.

### 2.8 RNA extraction and quantitative real‐time polymerase chain reaction (qRT‐PCR)

Total RNA was extracted with TRIzol from PC cells and tissues. All primers used for qRT‐PCR were designed and blasted in the National Center for Biotechnology Information database. RNA was reverse transcribed to cDNA and PCR was then performed using SYBR Green qPCR Master Mix (GenePharma, China) in triplicate. Human β‐actin was used as the internal control for mRNA expression. The primer sequences are shown in Table S1.

### 2.9 CCK‐8 proliferation assay

CCK‐8 kit (Dojindo Laboratories, Japan) was used to detect the proliferation ability of PC cells. Transfected cells were seeded in 96‐well plates at a concentration of 5 × 10^3^ cells per well. After cultured for 0, 24, 48, 72 and 96 h under the same conditions, PC cells were incubated with diluted CCK‐8 reagent following the instructions. Then, the absorbance was measured in a microplate reader at a wavelength of 450 nm.

### 2.10 EdU assay

An EdU kit (RiboBio, China) was used to detect the cell proliferation capability. In brief, after stable transfection for 48 h, cells were incubated with 5 μM EdU reagent diluted with DMEM for 3 h. Then, cells were permeabilized with 0.5% Triton X‐100 for 20 minutes prior to fixation with 4% paraformaldehyde for 30 minutes. Apollo and DAPI dyes were used to stain DNA and then nuclei. Images of EdU‐ and DAPI‐positive cells were acquired under a fluorescence microscope.

### 2.11 Colony formation assay

To verify colony formation ability, transfected cells were seeded into 6‐well plates at a density of 5000 cells per well and distributed evenly by pipetting. After 14 days, the cells were washed with PBS twice, fixed with 4% paraformaldehyde for 30 min and stained with 1% crystal violet for 30 min. Eventually, the cells were imaged and the colonies were counted.

### 2.12 Transwell invasion assay

To evaluate the cell migration ability, cells were cultured in serum‐free medium in the upper chamber of a Transwell plate (Corning, USA), and 700 μl of DMEM containing 10% FBS was added to the lower chamber. After 48 h, the cells were fixed and stained with crystal violet for 20 min, and the cells migrating through the upper chamber membrane were counted. Images were acquired by microscopy.

### 2.13 Luciferase reporter assay

The B3GNT6 fragment containing the possible m6A modification site and the corresponding mutated fragment were synthesized and cloned into the pmirGLO vector (Promega, USA) named B3GNT6‐WT and B3GNT6‐Mut. The synthesized vectors were separately co‐transfected with IGF2BP2‐si or si‐NC into 293 T cells. After co‐incubation for 24 h, cells were collected, and luciferase activities were measured with a Dual Luciferase Reporter Assay kit (GenePharma, China).

### 2.14 m6A IP and IGF2BP2 IP assays

Immunoprecipitation assays of m6A and IG2BP2 were performed with an RIP kit (BersinBio Biotech, China). In brief, approximately 1 × 10^7^ treated cells were collected and lysed for 30 min. DNA impurities were removed with DNase, the supernatant was collected after centrifugation, and the supernatant was then incubated with anti‐m6A and anti‐IGF2BP2 primary antibodies overnight at 4°C in a vertical orientation. Then, magnetic beads were added to incubate for 1 h. Finally, RNA was extracted, and target gene expression was detected by qRT‐PCR.

### 2.15 3‐DAA demethylation treatment

The overall methylation inhibitor 3‐DAA (Cayman, USA) was used to inhibit RNA methylation levels. After inoculation, cells were treated with 10 ug/ml 3‐DAA for 48 hours, then cells were collected for protein analysis.

### 2.16 IHC analysis

Paraffin sections of 5 paired clinical pancreatic specimens were prepared and baked in an oven at 65°C for 30 min. After routine dewaxing, hydration and antigen repair, sections were sealed with 5% goat serum for 30 min, placed in a wet box and incubated overnight with the IGF2BP2 primary antibody (ID:OTI3C8, OriGene, American) at 4°C. The next day, sections were incubated with the secondary antibody for 30 min, incubated with DAB for color development for 3 min and stained with hematoxylin for 3 min. Sections were sealed with neutral gum, and images were acquired with a microscope.

### 2.17 IF detection assay

After dewaxing, hydration and antigen repair, paraffin sections were blocked with immunostaining blocking solution for 1 h, incubated with IGF2BP2 and B3GNT6 (ID:21291‐1‐AP, Proteintech, American) primary antibody at 4°C overnight and secondary antibody for 1 h. After counterstaining with DAPI, sections were sealed with an anti‐fluorescence quenching agent and imaged under a fluorescence microscope. Treated PC cells were prepared as cell slides and subsequent experiments were performed as described above.

### 2.18 Western blot analysis

We used a RIPA mixture containing 1% PMSF lysate to lyse and obtain total protein from transfected cells. A BCA protein detection kit (Thermo Scientific Pierce, USA) was used to determine the protein concentration. Protein separation under 80 V, 30 min and 120 V, 1 h electrophoresis conditions, followed by transferring to polyvinylidene difluoride membranes at 200 mA for 1.5 h. Then, membranes were blocked with 5% skim milk and incubated with IGF2BP2 primary antibody (OriGene, American) and B3GNT6 (Proteintech, American) primary antibody overnight at 4°C. After washing, immunoreactions were visualized with an electroluminescence detection system.

### 2.19 Animal experiment

To evaluate the effect of IGF2BP2 on the tumor formation ability in vivo, 2 × 10^6^ cells were injected into the axilla of nude mice for subcutaneous tumor formation. After 9 days, modified IGF2BP2‐si or si‐NC was injected into the tumors every 3 days for 3 weeks. Tumor size was measured every 3 days. Finally, the subcutaneous tumors were excised and weighed to compare the tumor sizes, and protein expression was detected by immunohistochemistry and immunofluorescence. The care of laboratory animals was in accordance with the guidelines and ethical requirements of the Laboratory Animal Centre of Soochow University.

### 2.20 Statistical analysis

We used GraphPad 8.0 for charting. For statistical analysis, two‐tailed Student's t‐test between two groups were performed by SPSS Statistic 26 for windows. The Kaplan–Meier method was used for survival analysis. Data was reported as mean ± SD from three duplicate experiments. Results was considered statistically significant when *p* < 0.05 (*p < 0.05).

## 3 RESULTS

### 3.1 Overall expression pattern of m6A regulators

**FIGURE 1:**
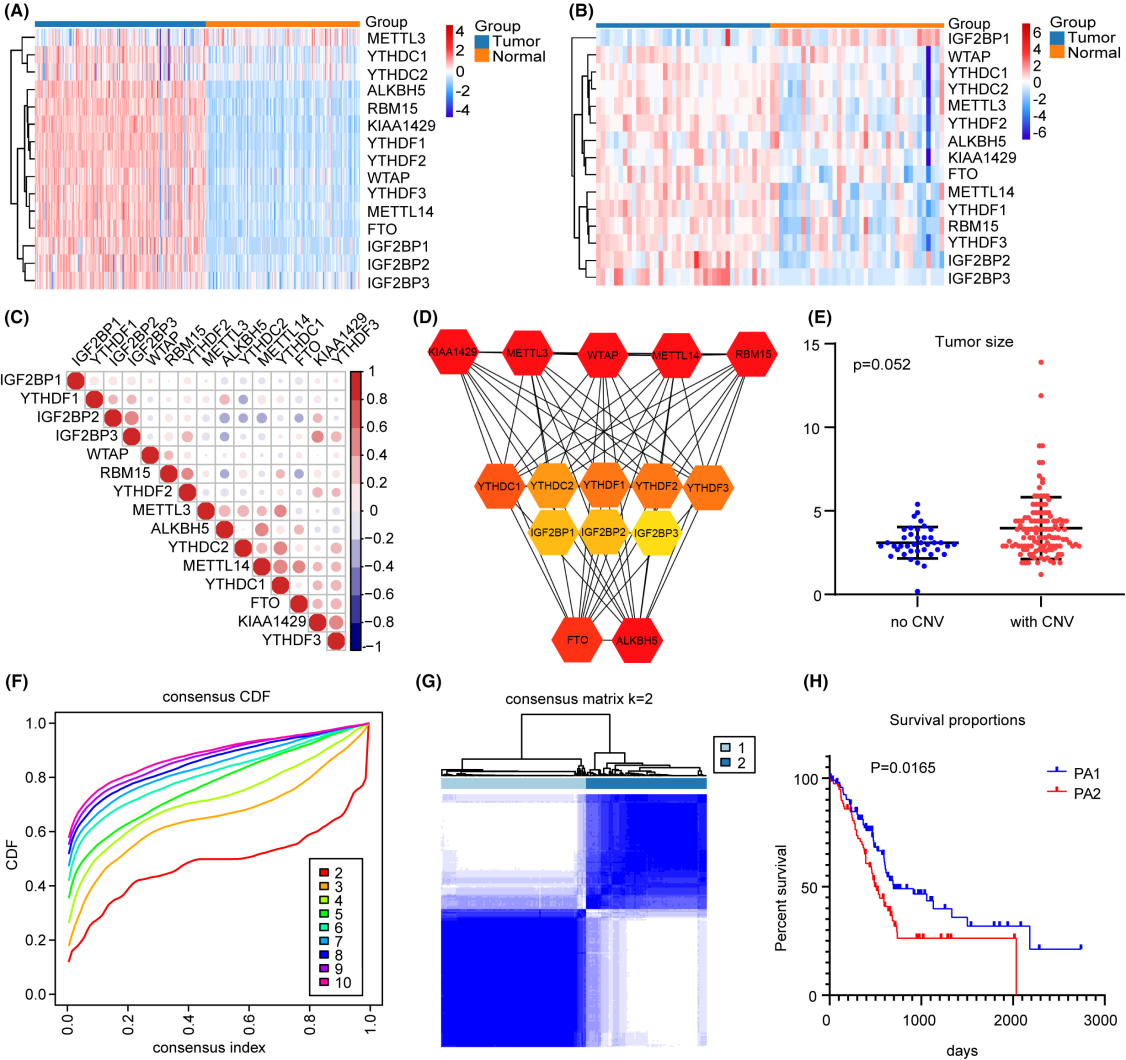
Expression of m6A methylation regulators and identification of consensus clusters by m6A regulators in PC. (A)–(B) Expression levels of 15 m6A RNA methylation regulators in PC in TCGA (A) and GEO (B) (red is up‐regulated and blue is down‐regulated). (C) Spearman correlation analysis of 15 m6A regulators in PC. (D) PPI network shows the interaction among 15 m6A regulators. (E) Relationship between overall CNV and tumor size. (F) Consensus clustering cumulative distribution function (CDF) for *k* = 2 to 10. (G) Consensus clustering matrix for *k* = 2. (H) Kaplan–Meier analysis for two subgroups.

Considering that methylation regulators perform different biological roles, we first analyzed the expression of 15 regulators in PC and found that compared with normal pancreatic expression profiles in the GTEx database, the regulators were generally upregulated in TCGA, it was also observed in the GEO database (GSE15471) (Figure 1A,B). Spearman correlation analysis showed a close relationship between the factors (Figure 1C). Analysis of the STRING database also revealed a positive correlation between multiple regulators (Figure 1D). Our CNV analysis showed tumor size with regulators CNV was bigger than that without CNV (Figure 1E), suggesting that CNV is an important element leading to the upregulation of regulators and the progression of PC.

### 3.2 Identification of two subgroups of PC by consensus clustering

To visualize the specific functions of m6A methylation regulators, we removed normal pancreatic samples and PC samples with no suitable clinical data, and used the ConsensusClusterPlus R package to perform consensus clustering. According to the expression similarity of the regulators, the cumulative distribution function (CDF) of our data seemed to be ideal when K = 2, and the PA1 (96 samples) and PA2 (80 samples) subgroups were then defined (Figure 1F,G). We compared the clinicopathological characteristics of PA1 and PA2 and found them to be consistent with the above expectations. K‐M survival analysis showed significant differences between the two subgroups (Figure 1H).

**FIGURE 2:**
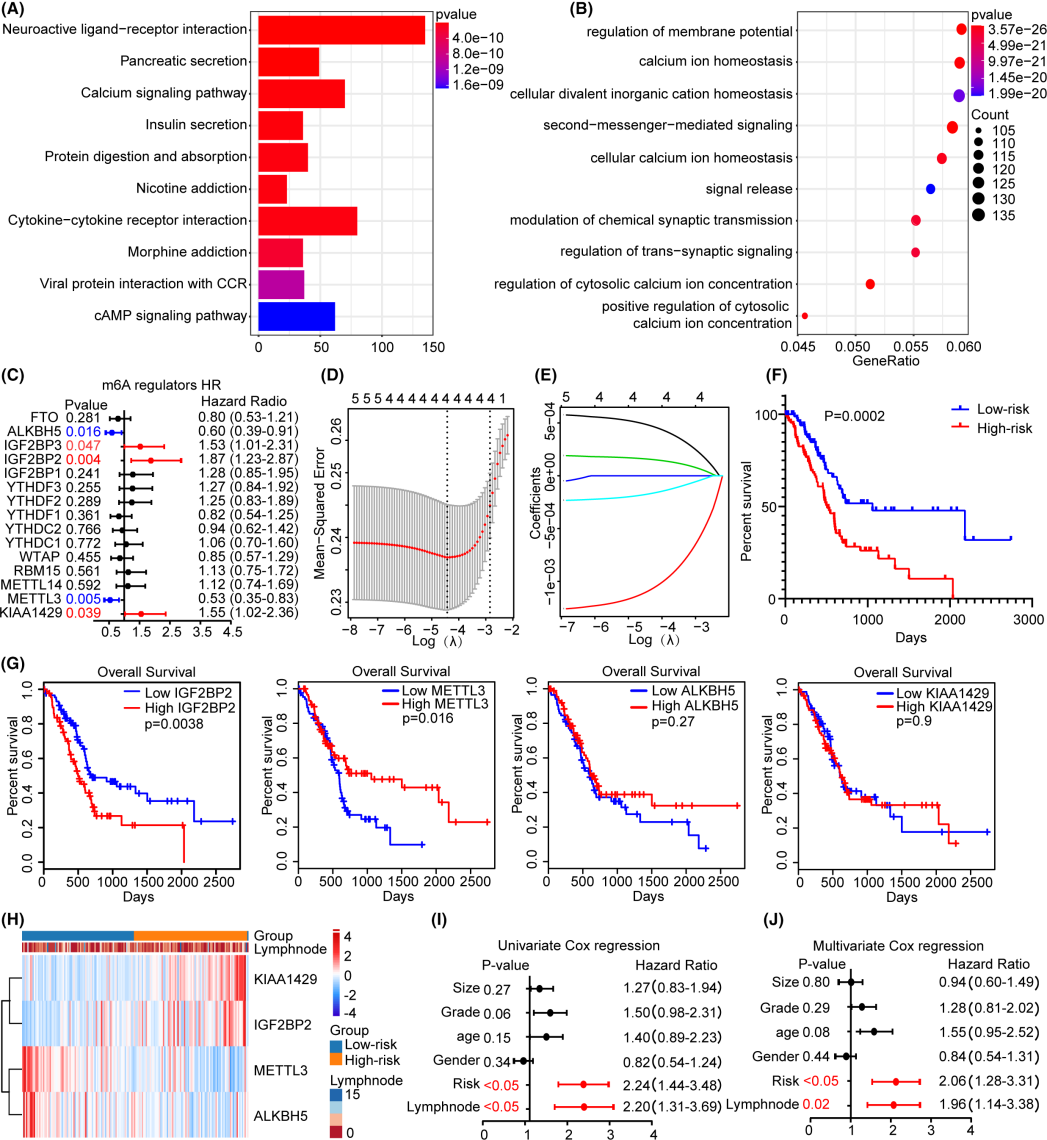
Enrichment analysis between subgroups and risk signature with four m6A RNA methylation regulators. (A–B) KEGG and GEO analysis of the subgroups. (C) The process of building the signature containing 15 m6A RNA methylation regulators. (D–E) The coefficients calculated by multivariate Cox regression using LASSO are shown. (F) Overall survival analysis between low‐ and high‐risk groups stratified by the risk score.(G) Overall survival analysis of the four m6A regulators. (H) The heatmap shows the expression of four m6A methylation regulators and related clinicopathological features in low‐ and high‐risk PC patients. (I–J) Univariate and multivariate Cox regression analyses of the association between clinicopathological factors and overall survival in TCGA.

The edgeR software package was used to analyze the difference between the expression profiles of PA1 and PA2, and the DEGs were selected based on the criteria |logFC| ≥ 1 and *p* ≤ 0.05. Finally, 2681 genes were identified, of which 724 genes were upregulated in PA1, and 1957 genes were upregulated in PA2. KEGG and GO enrichment analyses showed that the DEGs were mainly enriched in homeostasis or regulation of membrane potential (Figure 2A,B), such as Cytokine−cytokine receptor interaction and pancreatic secretion, indicating that mRNA methylation affects multiple biological processes.

### 3.3 Analysis of associations between m6A methylation regulators and clinicopathological characteristics

To further clarify the role of the regulators in survival, we conducted univariate Cox regression analysis. Among the 15 regulators, 5 were related to prognosis: IGF2BP2 (HR = 2.19), IGF2BP3 (HR = 1.53), METTL3 (HR = 0.53), ALKBH5 (HR = 0.6) and KIAA1429 (HR = 1.55) (Figure 2C). Survival models based on high reliability and LASSO regression are widely used to screen prognostic genes from high‐dimensional data; thus, we established risk characteristics and performed LASSO‐Cox regression analysis to calculate the risk score, and found that KIAA1429, METTL3, IGF2BP2 and ALKBH5 were the main contributors (Figure 2D,E), where the coefficient of KIAA1429, METTL3, IGF2BP2 and ALKBH5 were 0.00027, −0.00056, 0.00015 and −0.00019, respectively. According to the median risk score, low‐risk patients had higher survival status (Figure 2F). The respective survival rate analysis showed higher IGF2BP2 contributed to lower survival rate (Figure 2G). Heatmap showed that ALKBH5 and METTL3 were upregulated in the low‐risk group, while IGF2BP2 and KIAA1429 were upregulated in the high‐risk group (Figure 2H). We evaluated the correlations between the risk subgroups and the clinicopathological features. Univariate and multivariate Cox regression analyses showed that the risk score (*p* < 0.05) and lymph node metastasis (*p* < 0.05) were highly significant (Figure 2I,J). In summary, we believe that the accuracy of m6A methylation regulators for predicting the prognosis of PC was further corroborated. Considering that IGF2BP2 is highly expressed in PC, with a high HR, and is strongly independently associated with prognosis, we selected it as the main regulator for subsequent studies.

### 3.4 IGF2BP2 is a credible molecular prognostic marker in PC

**FIGURE 3:**
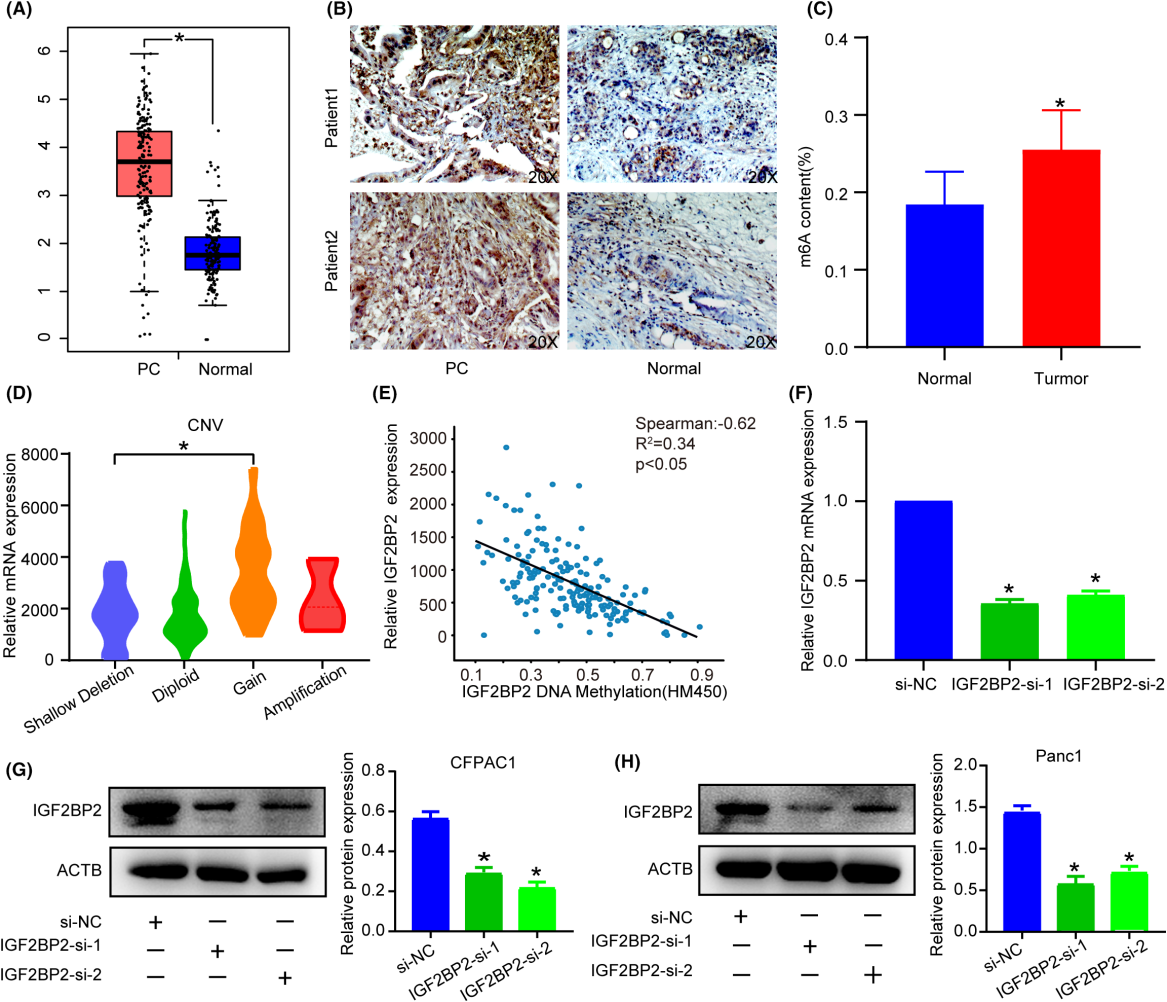
Expression of IGF2BP2 in pancreatic carcinoma. (A) Expression of IGF2BP2 mRNA in TCGA and GTEx database. (B) IHC (IGF2BP2)‐stained paraffin‐embedded sections verified the expression of IGF2BP2 protein in PC and normal tissue. (C) Quantification of overall m6A RNA methylation in PC tissues. (D) Relationship between different CNV types and IGF2BP2 expression level. (E) Relationship between IGF2BP2 DNA methylation and mRNA expression. (F) IGF2BP2 mRNA expression in si‐NC and IGF2BP2‐si groups. (G–H) IGF2BP2 protein expression in si‐NC and IGF2BP2‐si groups. (*p < 0.05).

TCGA and IHC analysis revealed high expression of IGF2BP2 in PC (Figure 3A,B). What's more, quantitative analysis of RNA methylation indicates an overall upregulation of m6A modification in PC tissues (Figure 3C). Combining positive experiments and bioinformatics results, we then sought to investigate some specific reasons for high IGF2BP2 and m6A expression. TCGA data analysis showed that increasing CNV contributed to a higher mRNA level (Figure 3D), while the DNA methylation level was negatively correlated with the mRNA expression (Figure 3E), indicating that DNA methylation and CNV were indispensable cooperative factors for high IGF2BP2 expression. These results indicated that IGF2BP2 was a marker of unfavorable prognosis in PC patients.

### 3.5 IGF2BP2 promotes PC cell proliferation and migration

**FIGURE 4:**
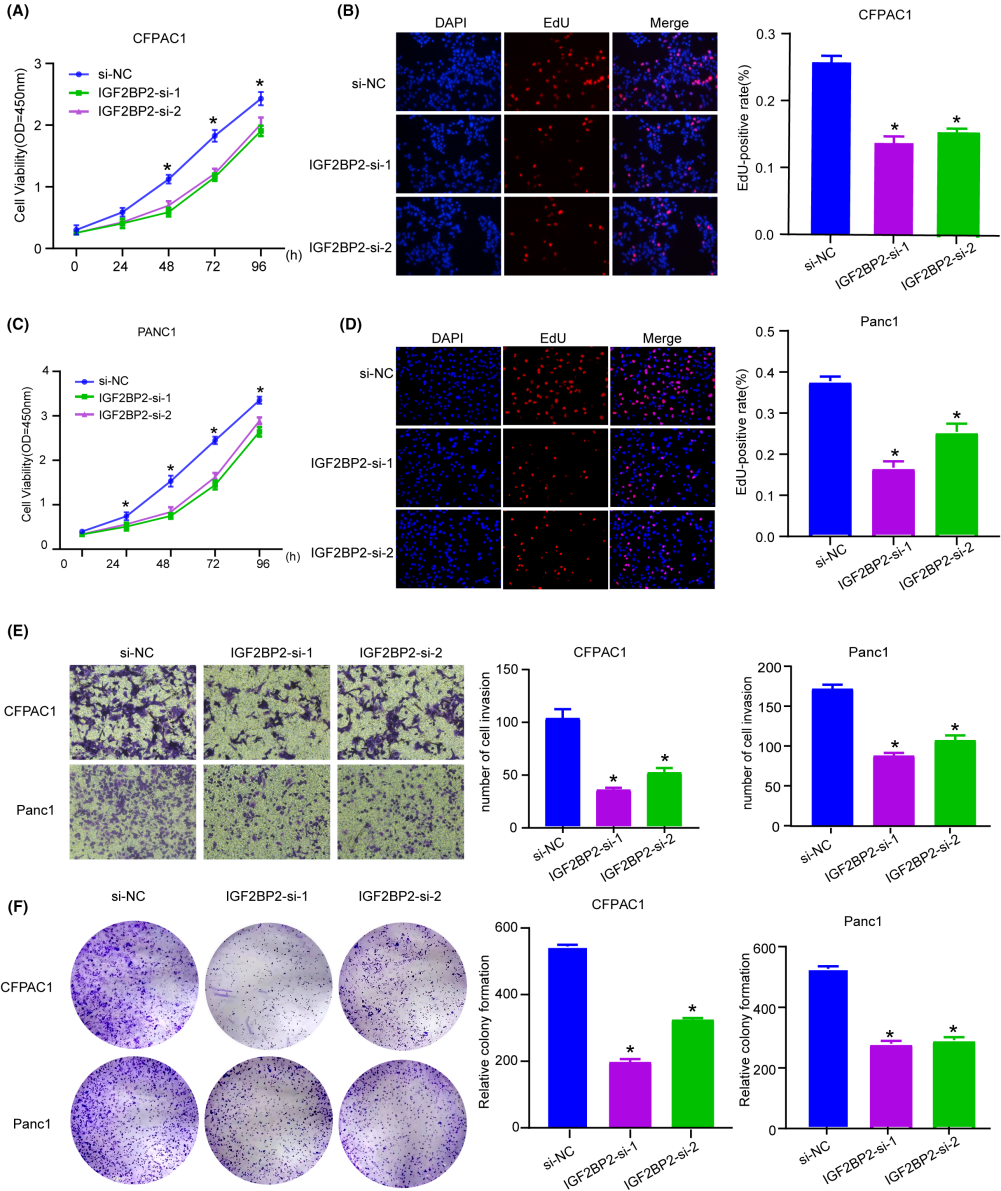
IGF2BP2 KO suppresses pancreatic carcinoma proliferation and migration ability. (A)–(D) CCK‐8 and EdU assay were used to detect cell proliferation ability. (E) Transwell experiment revealed PC cell migration ability with or without IGF2BP2 KO. (F) PC cell colony formation ability detection after IGF2BP2 KO. (*p < 0.05).

For further investigation of the biological function of IGF2BP2 in PC, we knocked down its expression in CFPAC1 and PANC1 cells (Figure 3F,H) and quantitative analysis of m6A revealed no changes (Figure S1). CCK‐8 and EdU assays indicated that silencing IGF2BP2 obviously suppressed the proliferation of PC cells (Figure 4A–D). Also IGF2BP2 KO decreased cell migration ability with Transwell assay (Figure 4E). The colony formation assay showed that inhibition of IGF2BP2 decreased cell viability (Figure 4F). These results illustrate that IGF2BP2 exerts oncogenic effects in PC.

### 3.6 IGF2BP2 regulates B3GNT6 expression

**FIGURE 5:**
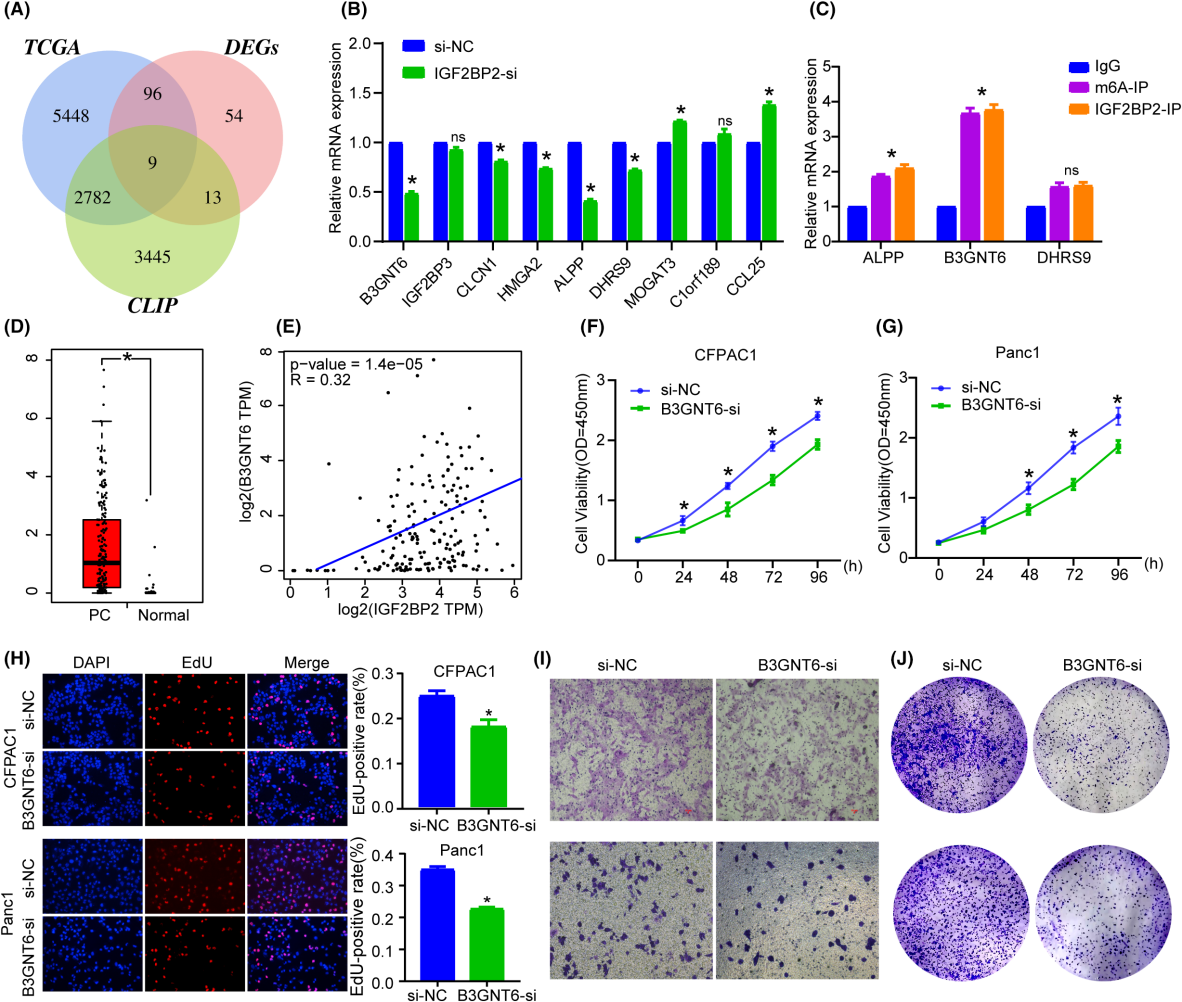
IGF2BP2 regulates B3GNT6 mRNA expression to promote pancreatic carcinoma. (A) Prediction of IGF2BP2 target genes, Venn diagram shows substantial and significant overlap among TCGA‐GTEx, IGF2BP2‐CLIP and DEGs between PA1 and PA2 subgroups. (B) Expression of predicted genes after IGF2BP2 KO. **C** m6A IP and IGF2BP2 IP verified the binding efficacy of target genes. (D) TCGA‐GTEx reveals the high expression of B3GNT6 in PC. (E) Spearman correlation analysis reveals positive co‐expression between IGF2BP2 and B3GNT6. (F–H) CCK‐8 and EdU assay revealed the PC cell proliferation ability after B3GNT6 KO. (I) Transwell experiment revealed PC cell migration ability after B3GNT6 KO. (J) B3GNT6 KO inhibited colony formation ability in PC cells. (*p < 0.05).

As an RNA binding protein, IGF2BP2 has been proven to bind to a variety of RNAs to regulate their expression. To identify potential target genes of IGF2BP2, we analyzed IGF2BP2 CLIP data and identified 6249 targets with relatively high associativity. Subsequently, PC patients were divided into high IGF2BP2 expression and low IGF2BP2 expression group, and differential gene expression analysis was conducted between the groups. In TCGA, we identified 8335 DEGs between PC and normal tissue, among which nine were common highly reliable genes (Figure 5A). qRT‐PCR confirmed that after knockdown of IGF2BP2, some genes were downregulated to varying degrees, of which, B3GNT6, DHRS9 and ALPP had the largest decreases (Figure 5B). We performed RIP with IGF2BP2 and m6A antibody (Figure S2) and results showed that B3GNT6 was more highly enriched than DHRS9 and ALPP (Figure 5C). Also, TCGA data analysis indicated that B3GNT6, ALPP and DHRS9 was upregulated in PC (Figure 5D; Figure S3). B3GNT6 is a beta‐1,3‐N‐acetylglucosaminyl transferase that adds an N‐acetylglucosamine moiety to N‐acetylgalactosamine‐modified serine or threonine residues to affect biosynthesis and metabolism. Studies have found that B3GNT6 is closely related to a variety of disease processes, such as immunity deficiency,^21^ colorectal cancer and m6A modification,^22^ Together, these results indicate that B3GNT6 may be the downstream target of IGF2BP2. Survival analysis with TCGA data showed high B3GNT6 was related to lower survival status, and Spearman correlation analysis also showed the co‐expression of IGF2BP2 and B3GNT6 (Figure 5E). Given that B3GNT6 is clinically relevant, closely related to m6A and understudied in PC, we mainly focused our research on it.

### 3.7 Inhibition of B3GNT6 decreased the proliferative ability of PC cells

We first knocked down B3GNT6 in cells, CCK‐8 and EdU incorporation assays indicated that silencing B3GNT6 overtly inhibited the proliferation of PC cells (Figure 5F–H). In addition, Transwell and colony formation assay showed that B3GNT6‐si inhibited cell migration and viability (Figure 5I,J). These results indicate that B3GNT6 can exert oncogenic effects in PC.

### 3.8 IGF2BP2 recognizes m6A methylation on B3GNT6 and increases its stability

**FIGURE 6:**
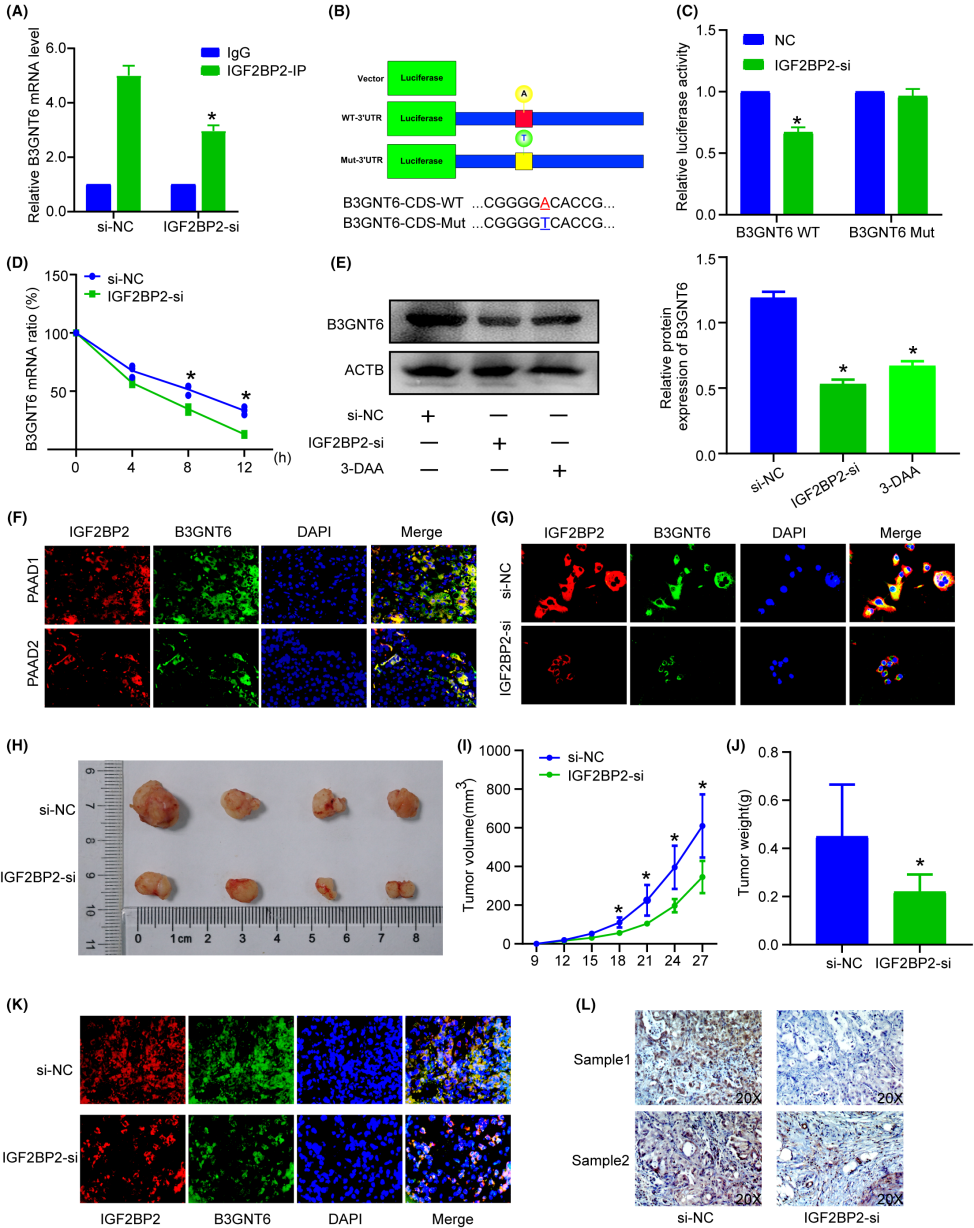
IGF2BP2 regulates B3GNT6 mRNA stability via an m6A‐dependent manner and promotes m6A content in PC. (A) IGF2BP2 IP verified B3GNT6 expression after IGF2BP2 KO. (B‐C) Dual luciferase reporter assays verified the methylation of B3GNT6 by IGF2BP2. (D) B3GNT6 mRNA stability in cells treated with IGF2BP2‐si. (E) B3GNT6 protein expression in cells after treated with IGF2BP2‐si and 3‐DAA. (F–G) Immunofluorescence staining confirmed the co‐expression of IGF2BP2 and B3GNT6 in PC tissues (F) and cells (G). (H–J) Detection of the tumorigenic ability of PC cells, including tumor size and tumor weight. (K) Immunofluorescence staining confirmed the co‐expression of IGF2BP2 and B3GNT6 in mice tumor tissue. (L) IHC(B3GNT6)‐stained paraffin‐embedded sections verified the low expression of B3GNT6 protein after IGF2BP2 KO. (*p < 0.05).

To further identify the underlying mechanism of IGF2BP2‐mediated B3GNT6 expression, we sought to determine whether and how IGF2BP2 interacts with B3GNT6 to affect its expression. We knockdown IGF2BP2 and RIP showed lower B3GNT6 was enriched (Figure 6A). Dual luciferase reporter assay was conducted and the results revealed that B3GNT6 was possibly recognized and regulated by m6A modification (Figure 6B,C).

Considering that IGF2BPs could regulate RNA stability, we sought to evaluate B3GNT6 mRNA stability. PANC1 cells were transfected with IGF2BP2‐si or the si‐NC, then treated with actinomycin D (Fdbio science, China) at 10 ng/ml at different time point (0, 4, 8 and 12 h). As shown, B3GNT6 mRNA decay rate in the IGF2BP2‐siRNA group was faster than that in the si‐NC group (Figure 6D), IGF2BP2‐KO and 3‐DAA could decreased B3GNT6 protein expression (Figure 6E), suggesting IGF2BP2 can increase B3GNT6 mRNA stability and contribute to the high B3GNT6 protein level in an m6A manner. IF staining revealed that IGF2BP2 and B3GNT6 had similar expression trends in PC tissue (Figure 6F), and knockdown of IGF2BP2 led to an obvious decrease in the B3GNT6 protein level (Figure 6G). Together, these results suggest that B3GNT6 could undergo m6A methylation, and IGF2BP2 functions as a reader of methylated B3GNT6 to increase its stability.

### 3.9 IGF2BP2 promotes tumorigenesis in vivo

**FIGURE 7:**
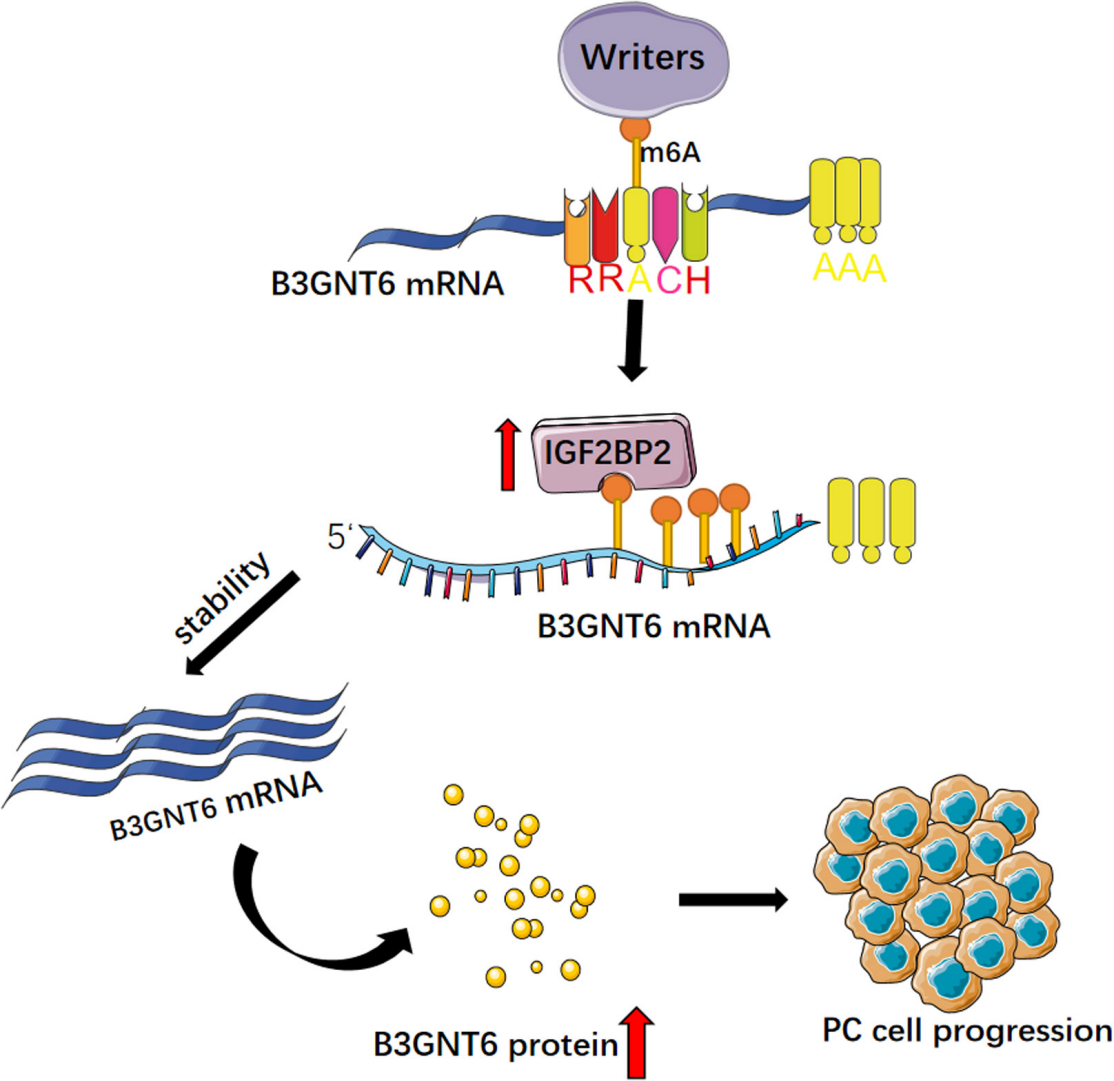
Mechanism diagram of IGF2BP2 promoting B3GNT6 expression via m6A methylation.

Subcutaneous tumorigenesis experiments in mice showed that knockdown of IGF2BP2 contributed to a decreased tumor size (Figure 6H,I) and tumor weight (Figure 6J), showing its significant ability in tumor formation. We evaluated the B3GNT6 expression level by IHC and IF, finding that knockdown of IGF2BP2 contributed to a lower B3GNT6 expression level (Figure 6K,L). Together, these data indicate that IGF2BP2 regulates B3GNT6 in an m6A manner and serves as an oncogene in PC (Figure 7).

## 4 DISCUSSION

We conducted a comprehensive expression profiling analysis of 15 methylation regulators and found overall high expression in PC samples. We divided PC samples into two subgroups and performed differential gene expression analysis. Then, we used LASSO‐Cox regression analysis to construct a methylation model and obtained risk score, finding that the survival rate in the high‐risk group was significantly lower than that in the low‐risk group. Finally, we identified the potential m6A regulator IGF2BP2 and downstream target gene B3GNT6. Subsequent in vivo and in vitro experiments were performed to verify the gene methylation modification. IGF2BP2 was proved to modulate cellular biological function through post‐transcriptional regulation and participate in the development and progression of cancers in an m6A manner, such as head and neck squamous carcinoma,^23^ thyroid cancer,^24^ colorectal cancer^25^ and macrophage phenotypic activation.^26^

The mortality of PC varies greatly in different regions. In addition, due to late detection and the scarcity of effective treatments, the survival rate is generally low.^27^ Currently, effective treatment for PC is still based on traditional surgical treatment, but still with a poor survival rate.^28^, ^29^ RNA methylation is the most common epigenetic modification, and m6A is the most universal methylation modification.^9^, ^30^ By modifying the methylation of specific sites in mRNA, one m6A methylation regulator may have different functions in different cancers.^31^

m6A modifies and regulates different aspects of mRNA, including its structure, stability, splicing, nuclear export, translation, decay, etc., and is also involved in cell fate determination, cell cycle regulation, and cell differentiation. Among the regulators, m6A writers mainly increase the level of methylation on RNA, which is the key step in m6A modification.^32^ As a reversible modification, m6A eraser has the opposite function of a writer to reduce the level of m6A modification, but may eventually contribute to similar functional results, because there are also m6A readers, a group of proteins that can recognize m6A,^33^, ^34^ thus, when writers or erasers cooperate with different readers, they can contribute to various biological functions, which also leads to the complexity of m6A modification.^35^

To date, m6A methylation has been found to function as a carcinogenic or tumor‐suppressive mechanism in glioblastoma,^36^ hepatocellular carcinoma,^37^, ^38^ and breast cancer,^39^ but the function of m6A methylation in PC is still unknown. Previous studies have shown that YTHDF2 inhibits adhesion, invasion, migration and EMT through YAP signaling.^40^ WTAP stabilizes Fak mRNA in PC to promote metastasis and gemcitabine resistance,^41^ PIK3CB m6A methylation promotes the progression of PTEN‐deficient PC by regulating the AKT signaling pathway,^42^ but the pattern of methylation and the progression of malignancy in PC remain to be explored. Our results show that KIAA1429, METTL3, IGF2BP2 and ALKBH5 contribute to the classification of PC and is closely related to the clinicopathological characteristics of PC. METTL3 was originally identified as a methyltransferase responsible for m6A modification,^43^ and growing evidence shows it functions in mRNA shearing,^44^ 3'‐UTR modification,^45^ translational regulation^34^ and decay.^31^ In contrast to YTHDF2's ability to promote mRNA attenuation,^36^ IGF2BP2 can promote the stability and maintenance of its target mRNA in an m6A‐dependent manner,^46^, ^47^ which is usually achieved after binding to an mRNA stabilizer.

B3GNT6 was identified as IGF2BP2 target in an m6A manner, and our research verified its oncogenic role in PC, considering that B3GNT6 is an essential enzyme for the synthesis of core 3 O‐glycan^48^ and to reduce malignant biological characteristics in colon, but was upregulated and a favorable prognostic factor in PC,^49^ we believed m6A played an essential role in glucose metabolism.

In theory, the reader, eraser and writer should have different expression patterns due to their different functions. However, our research found that the expression patterns of most regulators, whether promoting or inhibiting methylation, tended to be the same. We speculated that on one hand, there may be a proportional relationship. For example, the ratio of methylation/demethylation regulators exceeding a certain range will promote cancer or suppress cancer. On the other hand, different regulators have different affinities for the same gene and contributions to abnormal downstream activities caused by m6A methylation.^47^, ^50^ Third, there may be other molecules affecting m6A methylation modification, indeed, these still need to be further verified.

## 5 CONCLUSIONS

In summary, we have confirmed the elevated m6A level and revealed the significant effect of RNA methylation in PC. Besides we authenticate a novel gene IGF2BP2 is a credible marker and upregulated in PC, PCR and WB indicate that it promotes the B3GNT6 mRNA stability to contribute to the deterioration of PC, indicating that IGF2BP2 is a potential prognostic marker and therapeutic target in PC progression. Hence, our study provides some evidence for research of RNA methylation in PC.

## 6 ADVANTAGES AND LIMITATIONS

In this study, we first verified that m6A modification features play an important role in the development of PC through bioinformatics analysis. Combined with experiments, it was verified that IGF2BP2 can regulate B3GNT6 stability through m6A and may regulate the metabolic pathway of PC. However the deficiency is that we only carried out cell function experiments on B3GNT6 and no follow‐up pathway analysis. Clinically, only IHC analysis of specimens was performed. Due to insufficient number of specimens and insufficient follow‐up time, clinical research has not been carried out in depth. we will further expand the sample size for verification.

## AUTHORS' CONTRIBUTIONS

Xiaofeng Xue and Lei Qin contributed to the conception and design of the article. Pei Cao, Ding Sun and Weigang Zhang contributed to the acquisition of the data. Pei Cao and Yufan Wu contributed to the implementation of the experiment. Zuxiong Tang, Junyi Qiu and Ding Sun contributed to the acquisition of specimens. Pei Cao, Yufan Wu and Xiaofeng Xue contributed to the bioinformatics analysis. Xiaofeng Xue, Pei Cao and Junyi Qiu contributed to the analysis and interpretation of the data. Xiaofeng Xue, Lei Qin and Pei Cao contributed to the writing, review, and revision of the paper.

## FUNDING INFORMATION

This research was supported by the Youth Science Foundation of National Natural Science Foundation of China (No. 81802365).

## CONFLICT OF INTEREST

The authors declare no competing financial interests.

## ETHICS APPROVAL AND CONSENT TO PARTICIPATE

All animal experiments were performed according to the guide for the Laboratory Animal Centre of Soochow University. The care of laboratory animals was in accordance with the guidelines and ethical requirements of the Laboratory Animal Centre of Soochow University.

## CONSENT FOR PUBLICATION

All authors have read and approved the final manuscript.

## PUBLISHER'S NOTE

Springer Nature remains neutral with regard to jurisdictional claims in published maps and institutional affiliations.

## Supporting information


Figure S1

Figure S2

Figure S3

Table S1

## Data Availability

The experimental data that support this study are available upon request. Bioinformatics data was downloaded from TCGA and GEO database with R studio, the experimental data are all independently completed by the research group.
