# Supplementary figures and images for: IGF2BP2 promotes pancreatic carcinoma progression by enhancing the stability of B3GNT6 mRNA via m6A methylation
Source: Cancer Med. 2022 Jul 31;12(4):4405–20. doi: 10.1002/cam4.5096 (PMC9972174; doi:10.1002/cam4.5096)

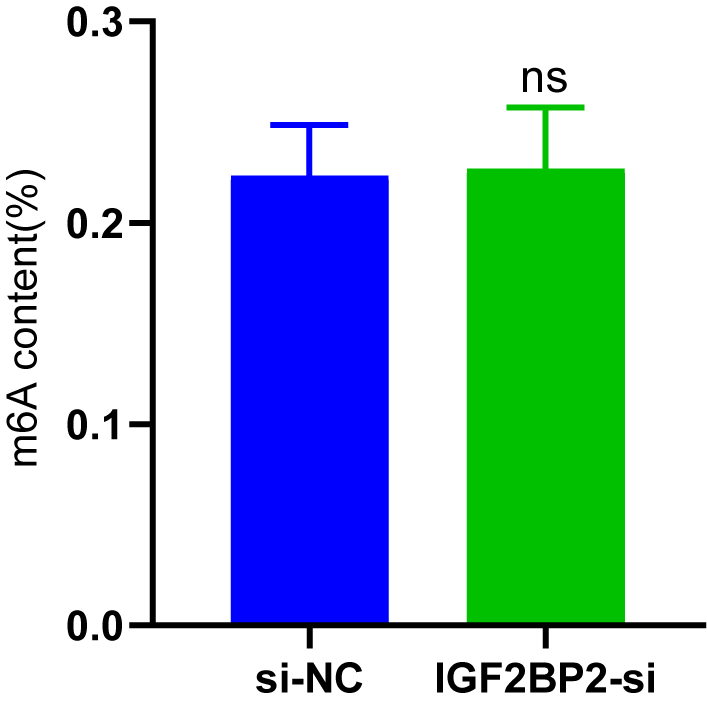

Supplement: Supplementary file 1 — Figure S1 [file CAM4-12-4405-s003.tif]

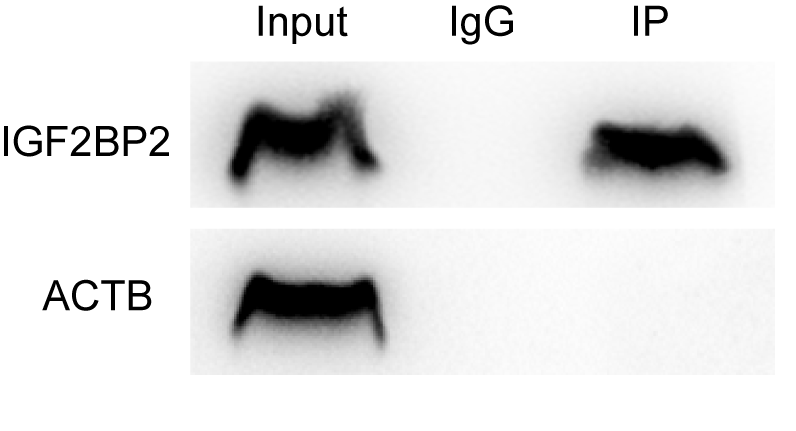

Supplement: Supplementary file 2 — Figure S2 [file CAM4-12-4405-s002.tif]

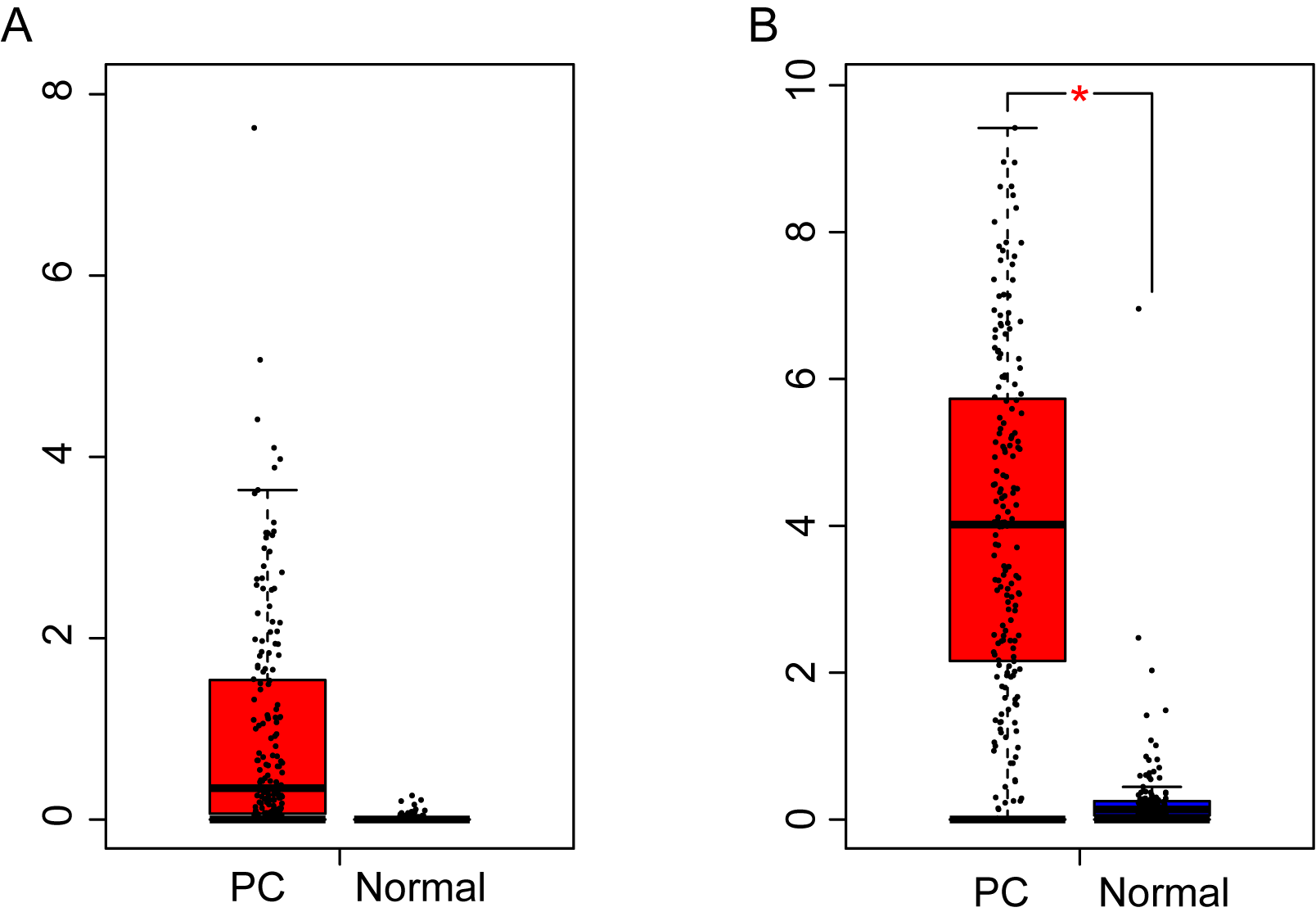

Supplement: Supplementary file 3 — Figure S3 [file CAM4-12-4405-s001.tif]
