# Supplementary material for: IGF2BP2 promotes pancreatic carcinoma progression by enhancing the stability of B3GNT6 mRNA via m6A methylation
Source: Cancer Med. 2022 Jul 31;12(4):4405–20. doi: 10.1002/cam4.5096 (PMC9972174; doi:10.1002/cam4.5096)
Supplement: Supplementary file 4 — Table S1 [file CAM4-12-4405-s004.docx]

Table S1. Primer sequence of mRNA or siRNA.

|  |  | Sequence |
| --- | --- | --- |
| IGF2BP2 | F | 5’-AGGGGACTTCTGAAGCATGC-3’ |
|  | R | 5’-ATCCTTTCGCTGACGTCTGG-3’ |
| CLCN1 | F | 5’-CCTTTGAACACTGCACCAGC-3’ |
|  | R | 5’-CGACAACCTTGGCCACAAAG-3’ |
| HMGA2 | F | 5’-ACCCAGGGGAAGACCCAAA-3’ |
|  | R | 5’-CCTCTTGGCCGTTTTTCTCCA-3’ |
| MOGAT3 | F | 5’-GTGGATAAGGAACCGGGCAA-3’ |
|  | R | 5’-GACCCCACAGCTTTCCTTGT-3’ |
| C1orf189 | F | 5’-ATGTCTGTGGAAAAGATGACAAAAGT-3’ |
|  | R | 5’-ATCTCTCTACATAAAAAGCTTTGCCC-3’ |
| ALPP | F | 5’-ACTGGGGCCTGAGATACCC-3’ |
|  | R | 5’-TCGTGTTGCACTGGTTAAAGC-3’ |
| IGF2BP3 | F | 5’-CCAAGCTAGACAAGCACTAGAC-3’ |
|  | R | 5’-GCGGCCATTTCATCAGGGA-3’ |
| B3GNT6 | F | 5’-TCAACCTCACGCTCAAGCAC-3’ |
|  | R | 5’-CAGGAAGCGGACTACGTTGG-3’ |
| CCL25 | F | 5’-ATGAACCTGTGGCTCCTGG-3’ |
|  | R | 5’-TGCTGCTGATGGGATTGCTA-3’ |
| DHRS9 | F | 5’-TCACCGACCCAGAGAATGTCAA-3’ |
|  | R | 5’-GCCGGGAACACCAGCATTATT-3’ |
| ACTB | F | 5’-TTGCTGACAGGATGCAGAAG-3’ |
|  | R | 5’-ACTCCTGCTTGCTGATCCACAT-3’ |
| IGF2BP2-si-1 | SS | 5’-CCCUCUCGGGUAAAGUGGAAUUGCATT-3’ |
|  | AS | 5’-UGCAAUUCCACUUUACCCGAGAGGGTT-3’ |
| IGF2BP2-si-2 | SS | 5’-GCUUGGUUGGAAGACUGAUUGGAAATT-3’ |
|  | AS | 5’-UUUCCAAUCAGUCUUCCAACCAAGCTT-3’ |
| B3GNT6-si | SS | 5’-UGGGCGUGAGUUUCUUAGCACUGCATT-3’ |
|  | AS | 5’-UGCAGUGCUAAGAAACUCACGCCCATT-3’ |
